# Supplementary material for: Lifetime and past-year suicidal behaviors among adolescents in Bangladesh: A two-stage stratified cluster sampling study
Source: Glob Ment Health (Camb). 2025 Feb 4;12:e24. doi: 10.1017/gmh.2025.10 (PMC11867828; doi:10.1017/gmh.2025.10)
Supplement: Al-Mamun et al. supplementary material [file S205442512500010Xsup001.docx]

**Supplemental Table 1.** Assessed Items and Response Options for PHQ-9, GAD-7, and ISI-2

| **Scale** | **Item** | **Response Options** |
| --- | --- | --- |
| **PHQ-9** | **Instruction:** The following items ask about common symptoms of depression. For each statement, select the option that best describes how often you have been bothered by the problem over the past two weeks. | |
|  | 1. Little interest or pleasure in doing things | 0 = Not at all, 1 = Several days, 2 = More than half the days, 3 = Nearly every day |
|  | 2. Feeling down, depressed, or hopeless | 0 = Not at all, 1 = Several days, 2 = More than half the days, 3 = Nearly every day |
|  | 3. Trouble falling or staying asleep, or sleeping too much | 0 = Not at all, 1 = Several days, 2 = More than half the days, 3 = Nearly every day |
|  | 4. Feeling tired or having little energy | 0 = Not at all, 1 = Several days, 2 = More than half the days, 3 = Nearly every day |
|  | 5. Poor appetite or overeating | 0 = Not at all, 1 = Several days, 2 = More than half the days, 3 = Nearly every day |
|  | 6. Feeling bad about yourself—or that you are a failure or have let yourself or your family down | 0 = Not at all, 1 = Several days, 2 = More than half the days, 3 = Nearly every day |
|  | 7. Trouble concentrating on things, such as reading the newspaper or watching television | 0 = Not at all, 1 = Several days, 2 = More than half the days, 3 = Nearly every day |
|  | 8. Moving or speaking so slowly that other people could have noticed? Or the opposite—being so fidgety or restless that you have been moving around a lot more than usual | 0 = Not at all, 1 = Several days, 2 = More than half the days, 3 = Nearly every day |
|  | 9. Thoughts that you would be better off dead or of hurting yourself in some way | 0 = Not at all, 1 = Several days, 2 = More than half the days, 3 = Nearly every day |
| **GAD-7** | **Instruction:** The following items ask about common symptoms of anxiety. For each statement, select the option that best describes how often you have been bothered by the problem over the past two weeks. | |
|  | 1. Feeling nervous, anxious, or on edge | 0 = Not at all, 1 = Several days, 2 = More than half the days, 3 = Nearly every day |
|  | 2. Not being able to stop or control worrying | 0 = Not at all, 1 = Several days, 2 = More than half the days, 3 = Nearly every day |
|  | 3. Worrying too much about different things | 0 = Not at all, 1 = Several days, 2 = More than half the days, 3 = Nearly every day |
|  | 4. Trouble relaxing | 0 = Not at all, 1 = Several days, 2 = More than half the days, 3 = Nearly every day |
|  | 5. Being so restless that it is hard to sit still | 0 = Not at all, 1 = Several days, 2 = More than half the days, 3 = Nearly every day |
|  | 6. Becoming easily annoyed or irritable | 0 = Not at all, 1 = Several days, 2 = More than half the days, 3 = Nearly every day |
|  | 7. Feeling afraid as if something awful might happen | 0 = Not at all, 1 = Several days, 2 = More than half the days, 3 = Nearly every day |
| **ISI-2** | **Instruction:** The following items ask about your sleep patterns and how they affect your daily life. Please select the option that best reflects your experiences over the past two weeks. | |
|  | 1. How satisfied/dissatisfied are you with your current sleep pattern? | 0 = Very satisfied, 1 = Satisfied, 2 = Neutral, 3 = Dissatisfied, 4 = Very dissatisfied |
|  | 2. To what extent do you consider your sleep problems to interfere with your daily functioning? (e.g., daytime fatigue, mood, ability to function at work/daily chores, concentration, memory, mood, etc.) | 0 = Not at all, 1 = A little, 2 = Somewhat, 3 = Much, 4 = Very much |
